# Intestinal Dysmotility and Associated Disorders in Intestinal Muscle of Methylglyoxal‐Treated Mice

**DOI:** 10.1111/nmo.70068

**Published:** 2025-05-02

**Authors:** Yuki Yamakawa, Taiki Mihara, Masatoshi Hori

**Affiliations:** ^1^ Department of Veterinary Pharmacology, Graduate School of Agriculture and Life Sciences The University of Tokyo Tokyo Japan

**Keywords:** inflammation, interstitial cells of Cajal, intestinal transit, methylglyoxal, peritoneal dialysis

## Abstract

**Background:**

Peritoneal dialysis (PD) is a renal replacement therapy approach to treat end‐stage renal failure. However, complications such as gastrointestinal dysmotility occur in patients undergoing PD, and the mechanisms underlying these complications have not been elucidated. We hypothesized that inflammation and dysfunction of the interstitial cells of Cajal (ICC) contribute to the PD‐induced gastrointestinal dysmotility.

**Methods:**

Mice were intraperitoneally administered a dialysate containing methylglyoxal (40 mM) every other day for 2 weeks to mimic the gastrointestinal complications in patients undergoing long‐term PD. The gastrointestinal transit capacity was evaluated using fluorescent dyes that were forcibly administered orally. To evaluate the inflammation and function of the ICC in the intestinal muscles, we performed real‐time polymerase chain reaction and immunohistochemical staining and measured spontaneous contractions ex vivo.

**Key Results:**

The intestinal transit capacity was significantly reduced in the methylglyoxal‐treated group compared to that in the control group. In the inflammatory evaluation, the number of neutrophils and macrophages in the intestinal muscles significantly increased in the methylglyoxal‐treated group compared to the control group. Moreover, the mRNA expression levels of *Tnf*, *Il1b*, and *Il6* were upregulated in the intestinal muscle from the methylglyoxal‐treated group. The mRNA expression of *Kit*, an interstitial cell of Cajal marker, was significantly decreased in the methylglyoxal‐treated group. In addition, the frequency of spontaneous contractions, an index of ICC function, was decreased in the methylglyoxal‐treated group.

**Conclusions and Inference:**

Our data suggest that the PD‐induced gastrointestinal dysmotility might be due to inflammation and dysfunction of the ICC in intestinal muscles.

## Graphical abstract

In patients undergoing long‐term peritoneal dialysis, intestinal transit capacity is often impaired, though the underlying mechanism remains unclear. Using a mouse model, we identified inflammation and impairment of interstitial cells of Cajal (ICC) in intestinal smooth muscle as potential contributors to this reduced transit capacity.

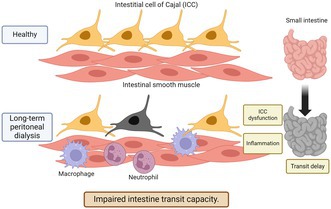

**Summary**

- Impaired gastrointestinal transit capacity was observed in methylglyoxal‐treated mice, a model mimicking long‐term peritoneal dialysis patients.
- Inflammation and dysfunction of Cajal interstitial cells were detected in the smooth muscle of the intestine in these mice.
- These findings suggest that gastrointestinal dysmotility frequently seen in peritoneal dialysis patients may be attributable to smooth muscle inflammation and Cajal cell dysfunction.

## 1 Introduction

Approximately 3.8 million individuals worldwide undergo dialysis for the symptomatic treatment of end‐stage kidney diseases [1]. There are two primary dialysis modalities, namely hemodialysis and peritoneal dialysis (PD). Notably, in the United States, the economic burden of PD is lower than that of hemodialysis [2]. Furthermore, PD offers several advantages over hemodialysis, such as improved quality of life and reduced incidence of depression‐ and cardiac disease‐related morbidities [3]. Despite these advantages and the government initiatives that aim at promoting PD, hemodialysis remains the most widely used approach in approximately 89% of the patients, whereas PD is performed in only 11% of the patients [4]. Additionally, there was a minimal shift toward the adoption of PD over the past few decades [5].

One of the primary factors contributing to the limited adoption of PD is the occurrence of gastrointestinal disorders as complications [6], observed in 57.9% of the patients undergoing PD [7], that reduce their overall quality of life [4]. Notable PD‐related gastrointestinal symptoms include constipation and delayed gastric emptying [6]. Although some reports suggest that the increased intra‐abdominal pressure associated with dialysate pooling causes gastrointestinal disorders [8], only a few have elucidated the detailed pathogenesis of gastrointestinal disorders in PD [9].

We previously reported that in the postoperative ileus, physical stimuli‐induced inflammation and interstitial cells of Cajal (ICC) dysfunction in the intestinal muscle layer resulted in reduced intestinal transit capacity, and suppression resulted in the restoration of the intestinal transit capacity [10, 11]. Therefore, we hypothesized that the PD‐related gastrointestinal disorders could be attributed to inflammation in the smooth muscle layer and ICC dysfunction.

## 2 Materials and Methods

### 2.1 Animals

Male *C57BL*/*6J* mice (wild‐type; Sankyo Labo Service, Tokyo, Japan) aged 8–11 weeks were used in this study. To exclude the possibility of sex‐related cycle‐induced alterations in the immune response, only male mice were used. The mice were co‐housed to minimize the occurrence of potential microbiome effects; they were bred under controlled conditions at 25°C and a 12‐h light/dark cycle with free access to water and feed (MF, Oriental Yeast Co., Ltd, Tokyo, Japan). Diet specifications are presented in Table S1. A Q‐Pla Tip (Sankyo Labo Service, Japan) was used for bedding, and TAR‐100E‐A (Toyo‐riko, Aichi, Japan) was used for the caging system. All interventions were performed during the light cycle between 8 am and 8 pm. All animal care and experimental procedures were performed in accordance with the Guidelines for Animal Use and Care as published by the University of Tokyo (Tokyo, Japan) and the International Animal Research: Reporting of In Vivo Experiments (ARRIVE) guidelines. All the experimental procedures were approved by the Institutional Review Board of the University of Tokyo (approval number: P23‐131).

### 2.2 Animal Model

The mice were treated with methylglyoxal (MGO; 40 mM) for 2 weeks to mimic the gastrointestinal complications in patients with long‐term PD. MIDPELIQ 250 (intraperitoneal; Terumo, Tokyo, Japan) was used as a vehicle. MGO‐treated mice models are widely used for the induction of peritoneal dysfunction caused by long‐term PD within a short time period [12]. Therefore, the MGO concentration and treatment duration were determined based on a previous report [13]. All experiments, except for the measurement of gastric emptying, were performed after euthanasia with deep anesthesia using isoflurane.

### 2.3 FITC Transit Test

Forty‐eight hours after the administration of the last dose, fluorescein isothiocyanate (FITC)‐dextran solution (100 μL; concentration, 5 mg/mL; Molecular weight: 70,000; Sigma, Tokyo, Japan; FD4‐100MG) was orally administered to the mice [14]. After 45 min, the mice were euthanized under deep isoflurane anesthesia, and the intestine was dissected starting from the stomach to the end of the colon. The intestine was divided into 15 sections: Sto, stomach; S1–S10, small intestine; Cec, cecum; and C1–C3, colon. The sections were finely cut in a physiological salt solution (NaCl, 136.9 mM; NaHCO_3_, 23.8 mM; glucose, 5.5 mM; KCl, 5.4 mM; CaCl_2_, 1.5 mM; MgCl_2_, 1.0 mM; and EDTA, 0.01 mM), shaken vigorously for 10 s, and centrifuged at 1500 × *g* for 10 min at 4°C. Each supernatant was transferred to a new tube and re‐centrifuged at 15,000 × *g* for 10 min at 4°C. A total volume of 200 μL of each resulting supernatant was transferred to a well of a 96‐well plate, and the fluorescence intensity (excitation wavelength, 498 nm; emission wavelength, 522 nm) was measured using an EMC‐418 plate reader (PerkinElmer Japan, Kanagawa, Japan, ARVO X2). The ratio of the fluorescence intensity of each section to the total fluorescence intensity of all sections was calculated. In addition, the geometric center (GC) of the FITC‐dextran distribution was calculated using the following equation: GC = sum (fluorescence intensity ratio [%] × section number)/100.

### 2.4 ^13^C‐Octanoic Acid Breath Test

Gastric emptying was evaluated using the ^13^C‐octanoic acid breath test as previously reported [15]. The animals were fasted for 12–16 h and placed in a chamber that was sufficiently large to allow them to move freely. After the administration of 200 mg of a test meal consisting of heated egg yolk and 0.2 μL ^13^C‐octanoic acid (Cambridge Isotope Laboratories Inc., MA, USA, CLM‐293‐1), a blow pump device (Thermo Fisher Scientific Inc., Tokyo, Japan) collected breath samples that accumulated in the chamber at a flow rate of 70 mL/min for a duration of 1 min; the samples were directed into a breath collection bag (Otsuka Pharmaceutical, Tokyo, Japan). After the administration of the test meal, the breath samples were collected every 10 min for a total duration of 120 min, then at 140, 160, 180, 210, and 240 min. The ^13^CO_2_/^12^CO_2_ ratio in the breath samples was analyzed using an infrared spectroscopic analyzer (Otsuka Electronics Co. Ltd., Osaka, Japan), and changes in ^13^CO_2_ (Δ^13^C, ‰) were calculated from the ^13^CO_2_/^12^CO_2_ ratio. A gas mixture composed of 5% ^12^CO_2_ and 95% O_2_ was used as the reference standard. The maximum concentration (*C*_max_; ‰), time to reach maximum concentration (*T*_max_; min), and area under the exhalation concentration‐time curve (AUC_240min_; ‰ ‧ min) were calculated using the Δ^13^C value. The half‐life (*T*_1/2_; min) was calculated from the elimination phase slope in the Δ^13^C curve [16].

### 2.5 Colon Transit

To evaluate the colonic transit ability of mice in vivo, we used glass beads and measured the excretion time, as described in a previous study [17]. Briefly, before testing, the mice were placed in individual small cages to induce feces excretion over 30 min. Under isoflurane anesthesia, glass beads (Cellpoint Scientific Inc., Gaithersburg, MD, USA, Cat# 5‐1455) with a diameter of 1.5 mm were inserted into the colonic flexure, approximately 3 cm from the anus, using a 20G stainless steel probe. During induction and maintenance, 2% and 1.5% isoflurane were, respectively, administered at a flow rate of 1 L/min. After insertion, the mice were returned to individual cages, and the time from the onset of the righting reflex to the excretion of the glass beads was recorded.

### 2.6 Whole‐Mount Immunohistochemistry

The following physiological saline solution was used for immunohistochemistry of the muscle layer from the duodenum, jejunum, and ileum (NaCl, 136.9 mM; NaHCO_3_, 23.8 mM; glucose, 5.5 mM; KCl, 5.4 mM; CaCl_2_, 1.5 mM; MgCl_2_, 1.0 mM; and EDTA, 0.01 mM). The mice were euthanized under deep isoflurane anesthesia 48 h after the administration of the last dose. The isolated intestine was opened along the mesenteric attachment and the mucosal and submucosal layers were removed using incision scissors and tweezers. The smooth muscle layer of the intestine is named the whole‐mount specimen. The specimens were fixed in acetone and methanol for 10 min on ice. Afterwards, they were washed three times in Tris‐buffered saline (TBS; Tris–HCl, 0.05 M and NaCl, 0.15 M), blocked using 2% bovine serum albumin (Funakoshi, Tokyo, Japan, PRO‐422) in TBS for 30 min, and incubated overnight at 4°C with primary antibodies. The muscle layers were then washed three times and incubated with secondary antibodies at 25°C for 1 h. Finally, they were washed three times with TBS and observed under a confocal microscope (LSM 700, Zeiss Japan, Tokyo, Japan). The cluster of differentiation (CD) 68‐positive cells in the enteric plexus layer were counted at four randomly selected locations in each sample and the mean value was calculated. The CD68, CD117 (c‐kit), platelet‐derived growth factor receptor α (PDGFRα), and PGP9.5 positive areas in the enteric plexus layer were measured. The enteric plexus was defined as the layer with PGP9.5 positive nerves. The used primary and secondary antibodies are listed in Table S2.

### 2.7 Myeloperoxidase Staining

Whole‐mount preparations were fixed using 10% neutral‐buffered formalin for 30 min and washed three times using TBS. The preparations were incubated for 5 min in 10 mL of TBS containing 10 mg of Hanker‐Yates reagent (Polysciences, Warrington, PA, USA, 08661‐5) and 10 μL of 30%–35.5% hydrogen peroxidase (Mitsubishi Gas Chemical Co., Tokyo, Japan, 081‐04215); then, they were washed with TBS for at least 10 min. The myeloperoxidase (MPO)‐positive neutrophils were counted under a microscope (OLYMPUS, Tokyo, Japan; SZ‐ST) in four randomly selected areas in each preparation.

### 2.8 Real‐Time Polymerase Chain Reaction (RT‐PCR)

Total RNA was extracted from the intestinal smooth muscle using TRIzol reagent following the manufacturer's instructions and reverse‐transcribed using ReverTra Ace (TOYOBO, Osaka, Japan, FSQ‐201) and random primers (TOYOBO, Osaka, Japan, FSK‐301). The cDNA was denatured at 95°C for 1 min and amplified through 40 cycles at 95°C for 15 s and 60°C for 1 min using SYBR Green (TOYOBO, Osaka, Japan, QPK‐212). The primer sets used in this study are listed in Table S3.

### 2.9 Intestinal Contraction Experiment

The whole‐mount preparations were placed under a resting tension of 10 mN. The isometric force was measured using a force transducer (Orientec, Tokyo, Japan, T7‐30‐230) and the data amplified using a strain amplifier (Yokogawa, Tokyo, Japan, AS1201) were recorded in a pen recorder (Yokogawa, Musashino, Japan, 3056) [18]. The strips were allowed to equilibrate for at least 20 min. Afterwards, tetrodotoxin (TTX; Wako, Osaka, Japan, 206‐11071) was added at a final concentration of 1 mM. After 20 min, the frequency of spontaneous contractions was measured again for 1 min (Figure S4). Spontaneous contractions were obtained as waveforms, and the number of maxima within 1 min in the waveform was defined as the frequency.

### 2.10 Statistical Analyses

All the results are presented as scatter plots with the mean and standard error of the mean (SEM). The data were statistically analyzed using Student's *t*‐test for comparisons between two groups and one‐way ANOVA followed by Holm–Sidak test for comparisons among three groups. Results having a *p‐*value < 0.05 were considered statistically significant. All statistical analyses were performed using GraphPad Prism software (version 9; San Diego, CA, USA).

## 3 Results

### 3.1 Intestinal Transit Capacity Was Impaired in the MGO‐Treated Mice

Long‐term PD can cause various complications [8]. There had been no mouse model that mimics the pathology induced by long‐term PD in a clinically relevant manner. Recent research has shown that continuous administration of MGO could mimic these pathological conditions due to PD within a “short time (2–3 weeks) in mice”, whereas it typically takes a “long time (3‐5 years) in humans” to develop [12]. However, no previous studies reported the use of MGO‐treated mice as a model of PD‐induced gastrointestinal disorders. Therefore, this study firstly aimed at investigating whether the PD‐induced impairment of gastrointestinal transit caused by PD also occurs in the MGO‐treated mice.

**FIGURE 1:**
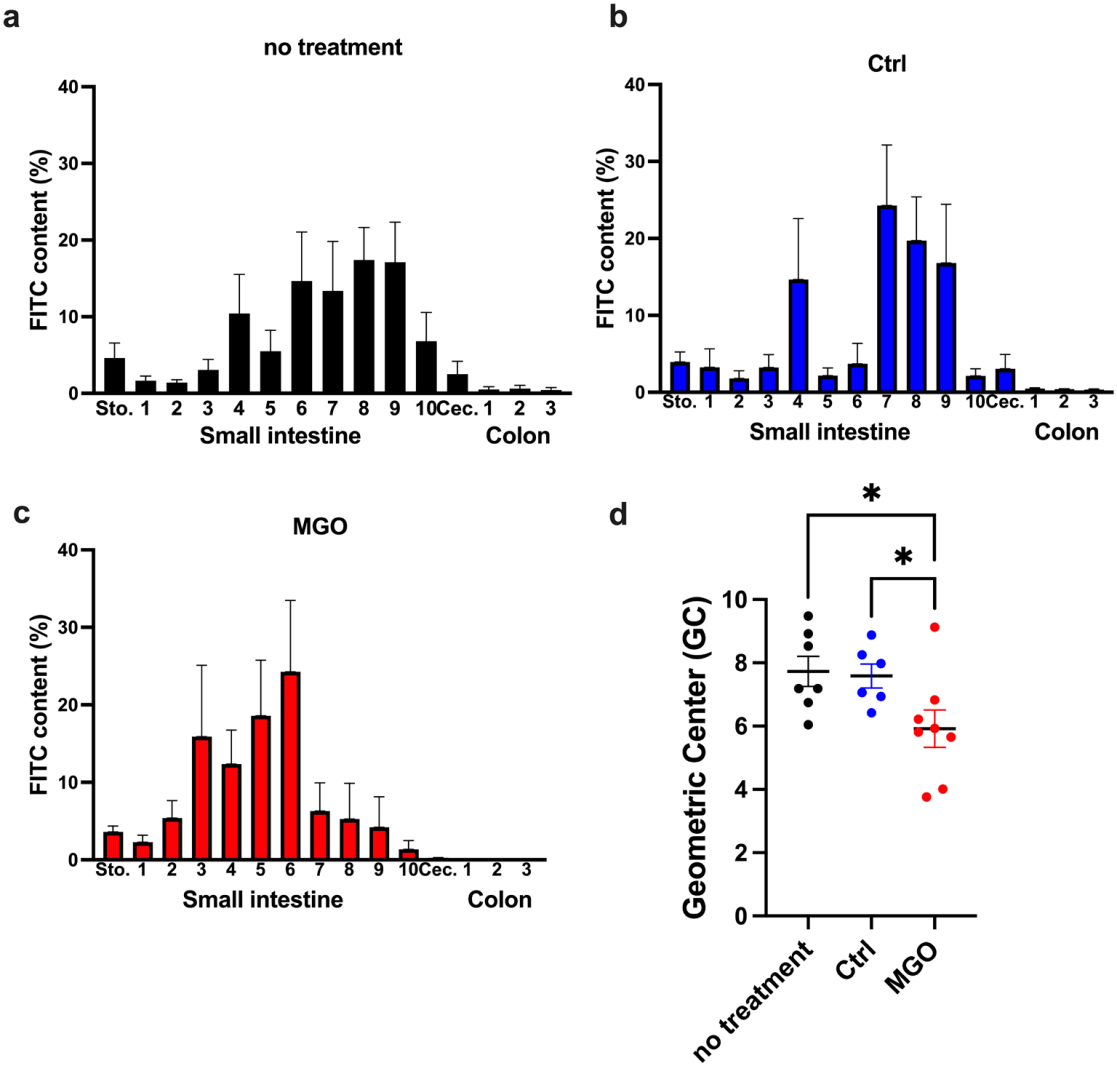
MGO administration is associated with reduced gastrointestinal transit capacity. (a–c) Fluorescein isothiocyanate (FITC) content (%) in each section 45 min after the oral administration of FITC solution. (d) Geometric centers calculated from (a) to (c). *p < 0.05 (*n* = 6–8). Each column shows mean ± SEM.

First, the intestinal transit capacity examined using the FITC transit test was compared between two groups of mice, namely the group that received no treatment and the dialysate group without MGO, to examine the dialysate effects on gastrointestinal transit (Figure 1a,b). Almost 60% of the FITC was located in sections S6–S9 in both groups, and there was no significant difference in the calculated GCs (Figure 1d), suggesting that the dialysate without MGO did not impair the intestinal transit capacity. Hereafter, the control group refers to the mice that were treated using the dialysate without MGO.

Next, we evaluated the intestinal transit capacity of the MGO‐treated mice. Unlike the untreated and dialysate without MGO treatment groups, 60% of the FITC was located in sections S3–S6 in the MGO group (Figure 1c). The GC content of the MGO‐treated mice was significantly lower than that of the control group, indicating an impaired gastrointestinal transit capacity in the MGO group (Figure 1d). These findings indicate that MGO‐treated mice can be used as a model for the PD‐induced impairment of the small intestine transit capacity.

### 3.2 Gastric Emptying Capacity and Colon Transit Were Not Impaired in the MGO‐Treated Mice

**FIGURE 2:**
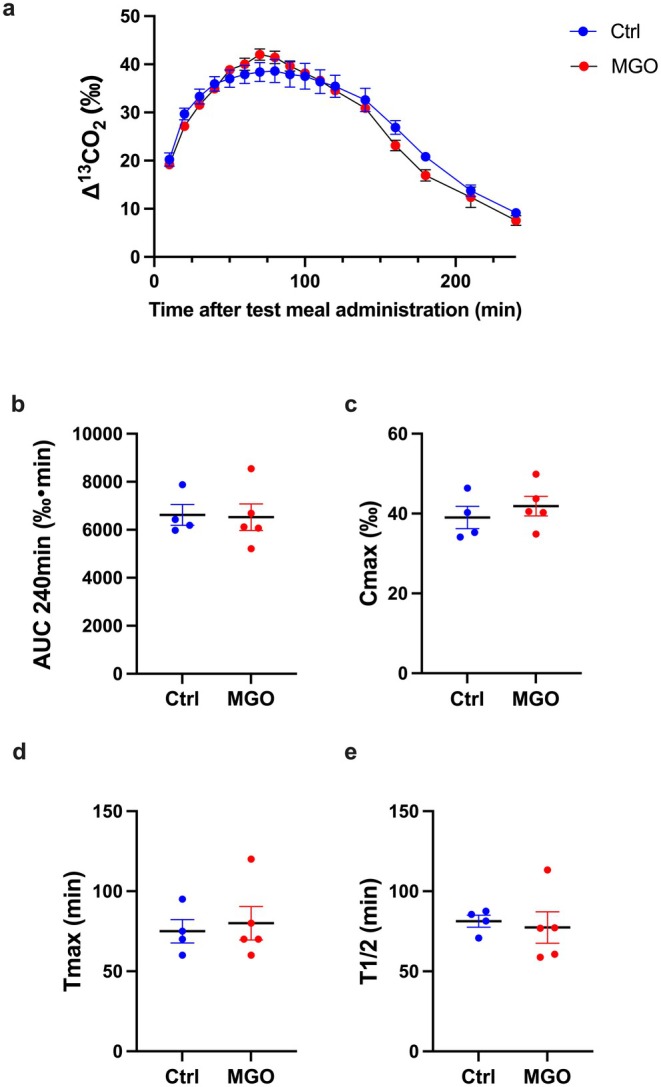
Normal gastric emptying capacity in methylglyoxal (MGO)‐treated mice. (a) Time course of gastric emptying measured using the ^13^C‐octanoic acid breath test in the control and MGO‐treated mice groups. Each column shows the mean ± SEM (*n* = 4–5). (b–e) Quantification of AUC240min, Cmax, Tmax, and T1/2 are calculated from (a). Each column shows the mean ± SEM (*n* = 4–5).

The gastric emptying capacity decreases in patients undergoing PD [7]. Thus, we investigated whether the MGO‐treated mice showed impaired gastric emptying capacity similar to the pattern observed in the patients undergoing PD. The ^13^C‐octanoic acid breath test was performed and various gastric emptying rate indices were measured. There were no significant differences in the ^13^CO_2_ emission dynamics (Figure 2a) or any of the calculated indices between the control and MGO‐treated mice groups (Figure 2b–e). These findings confirm that the MGO‐treated mice cannot be used as a model for the PD‐induced impairment of the gastric emptying capacity. As patients undergoing PD exhibit delayed colon transit [19], we also investigated whether MGO‐treated mice showed impaired colon transit; however, we found no significant differences (Figure S1). These findings suggest that the impaired transit capacity observed in Figure 1 could potentially be attributed to the small intestine.

### 3.3 Macrophages and Neutrophils Infiltrated Into the Intestinal Muscle Layer in the MGO‐Treated Mice

Inflammation decreases intestinal contractility [10]. Thus, we attempted to verify the presence of inflammation in the intestinal muscle layer due to intestinal dysmotility. Inflammation in the intestinal muscle layer was assessed based on the infiltration of CD68‐positive macrophages and neutrophils.

**FIGURE 3:**
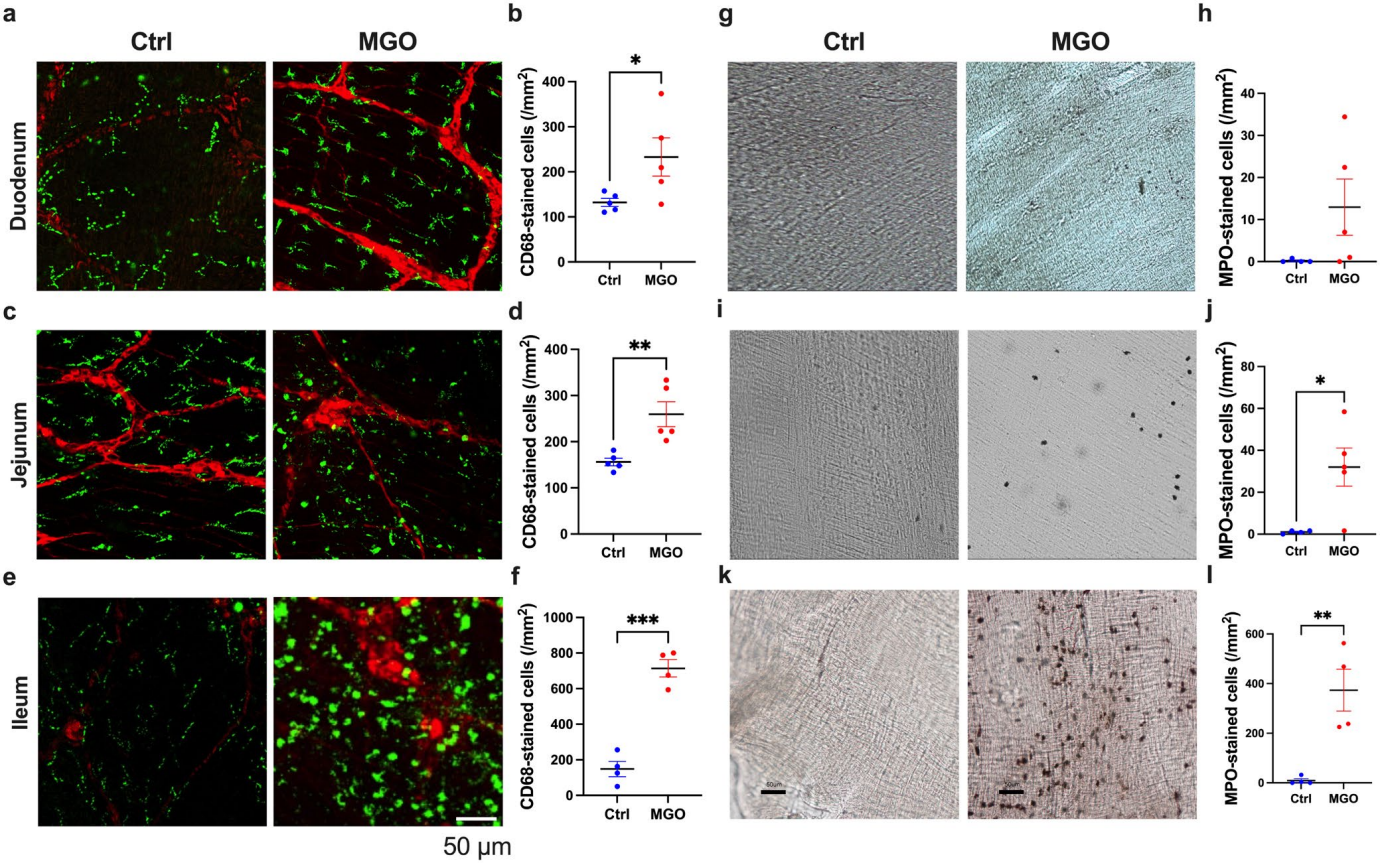
Inflammatory cell infiltration into the intestinal muscle layer from MGO‐treated mice. (a, c, e) Fluorescent immunostaining of the CD68‐positive macrophages in the smooth muscle layers of duodenum, jejunum, and ileum. The red and green signals indicate the PGP9.5‐positive enteric plexuses and CD68‐positive macrophages, respectively (white scale bar = 50 μm). (b, d, f) The number of CD68‐positive macrophages. (g, i, k) Images of myeloperoxidase (MPO) staining of the smooth muscle layers of the duodenum, jejunum, and ileum (black scale bars = 50 μm). (h, j, l) The number of neutrophils. *p < 0.05 (*n* = 4–5), **p < 0.01 (*n* = 4–6), and ***p < 0.001 (*n* = 4–6). Each column represents the mean ± SEM.

Immunohistochemical staining of the CD68‐positive macrophages revealed the presence of few resident dendritic macrophages in the control group (Figure 3a–f). In contrast, activated macrophages, having many formed rounds [20], significantly infiltrated the intestinal muscle layer of the duodenum, jejunum, and ileum in the MGO‐treated mice (Figure 3a–f). Almost no neutrophils were observed in the control group, whereas numerous neutrophils were detected in the intestinal smooth muscle layer of the ileum in the MGO‐treated mice (Figure 3g–l).

**FIGURE 4:**
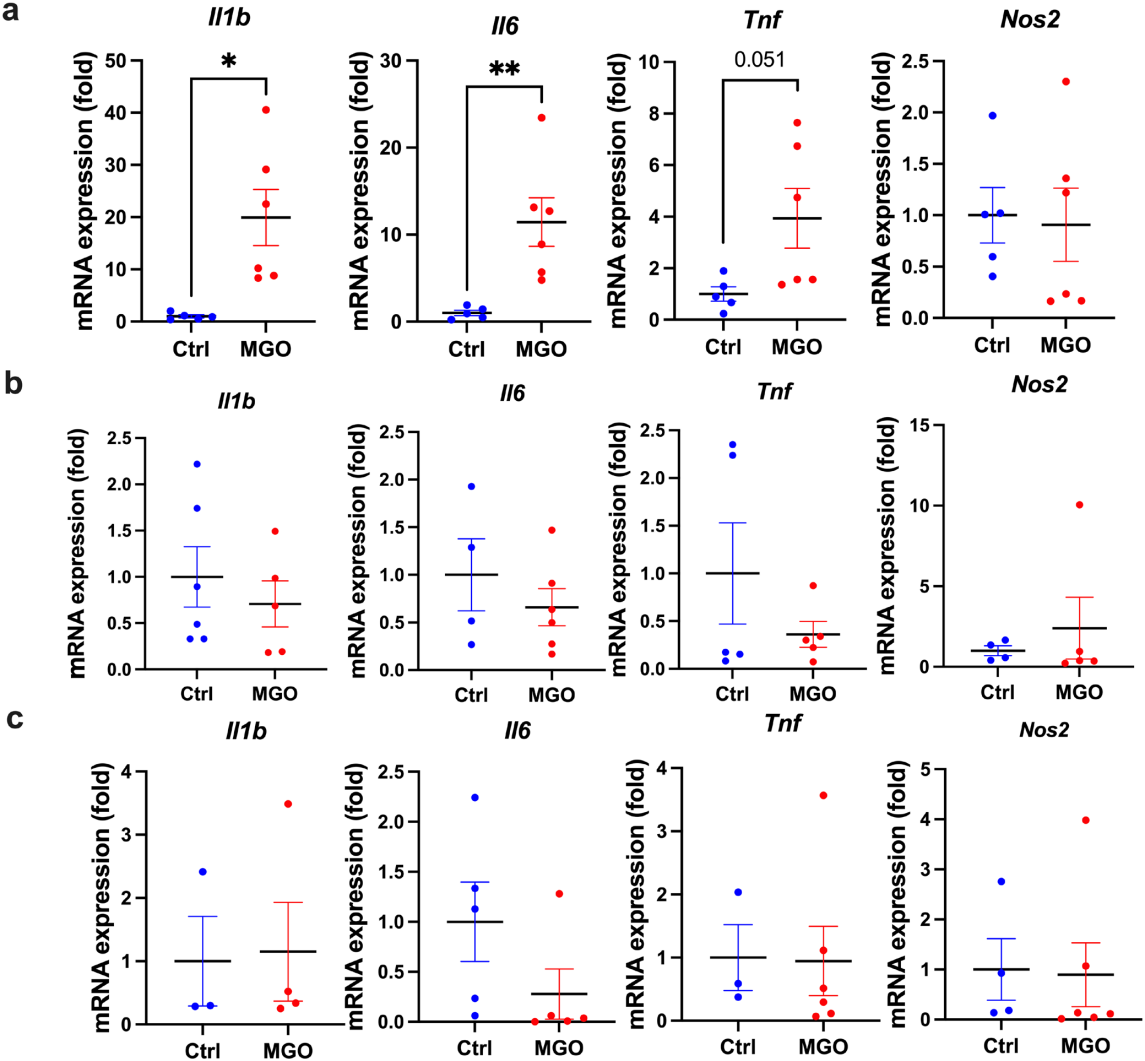
The mRNA expression of some markers in the intestinal muscle layers of the control and MGO‐treated mice. (a–c) MRNA expression of *Il1b, Il6, Tnf*, and *Nos2* at 3, 24, 48 h, respectively, after the administration of the last MGO dose. *p < 0.05 (*n* = 5–6); **p < 0.01 (*n* = 5–6). Each column represents the mean ± SEM.

Additionally, we measured the mRNA expression levels of some inflammatory mediators, such as *Il1b*, *Il6*, *Tnf*, and *Nos2*, in the intestinal smooth muscles at 3, 24, and 48 h after the administration of the last MGO dose. A significant upregulation was observed in the expression levels of *Il1b*, *Il6*, and *Tnf* transcripts in the MGO‐treated mice 3 h after the administration of the last MGO dose compared to those in the control mice (Figure 4a). This upregulation disappeared 24 and 48 h after the administration of the last MGO dose (Figure 4b,c). The increase in mRNA expression of pro‐inflammatory cytokines 3 h after MGO administration could be an artifact resulting from intraperitoneal administration. To rule out this possibility, we compared the mRNA expression of inflammatory mediators in the muscle layer of the ileum between “completely” no‐treated mice and vehicle‐only treated mice at 3 h post‐treatment. No differences were found (Figure S2), suggesting that the upregulation of mRNA expression caused by MGO treatment at 3 h (Figure 4a) was not an artifact of intraperitoneal administration.

These findings suggested that inflammation occurred in the intestinal muscle layer of the MGO‐treated mice.

### 3.4 *Kit* mRNA Expression Was Reduced in the Intestinal Muscle Layer of the MGO‐Treated Mice

**FIGURE 5:**
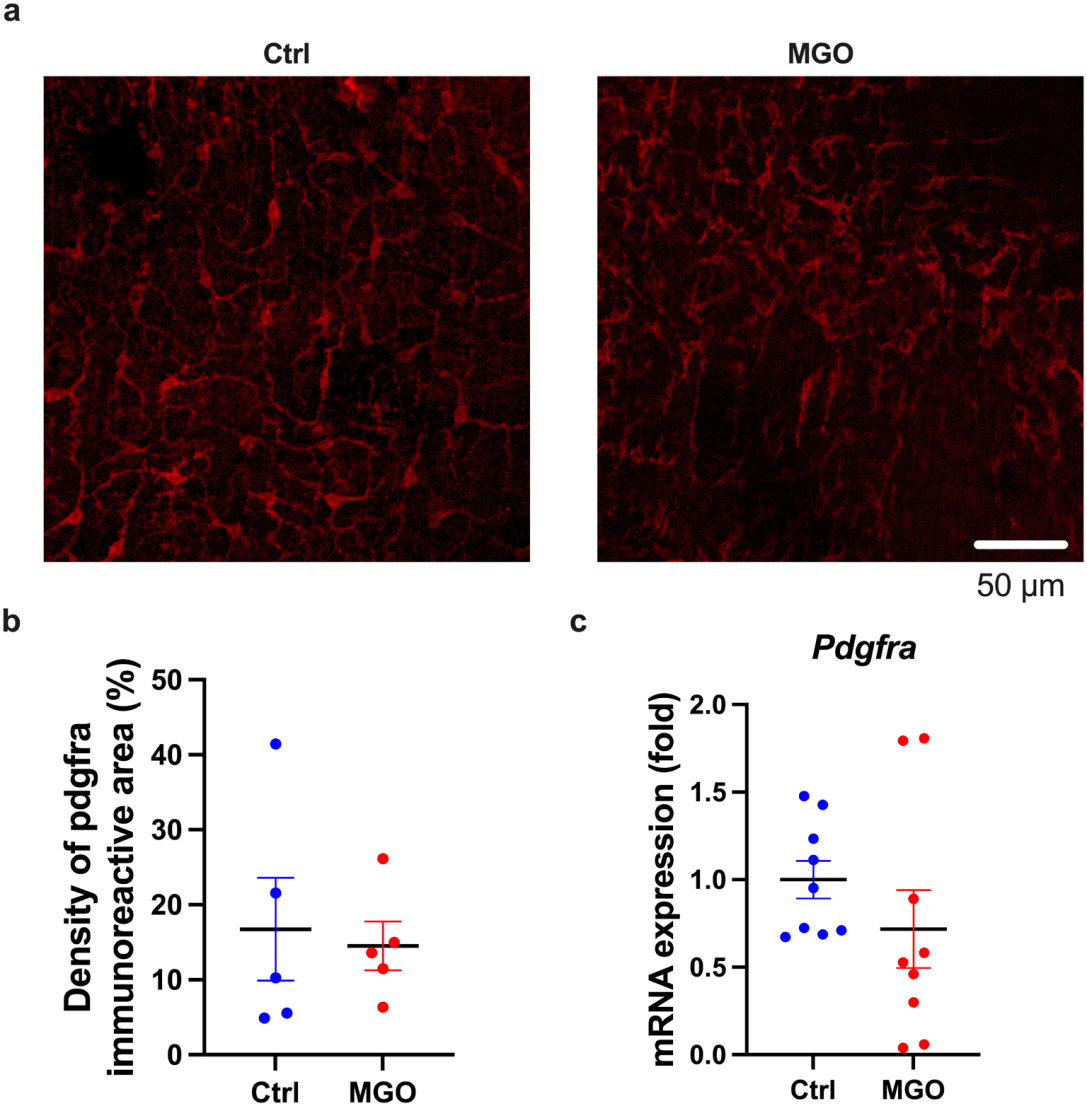
No change in the PDGFRα‐positive cells morphology and *Pdgfra* mRNA expression in the ileal muscle layer of MGO‐treated mice. (a) PDGFRα‐positive areas stained by immunofluorescent staining. The red signal indicates PDGFRα (white scale bar = 100 μm). (b) Density of the PDGFRα‐positive area. (c) RT‐PCR of the *Pdgfra* mRNA expression in the ileal muscularis.

**FIGURE 6:**
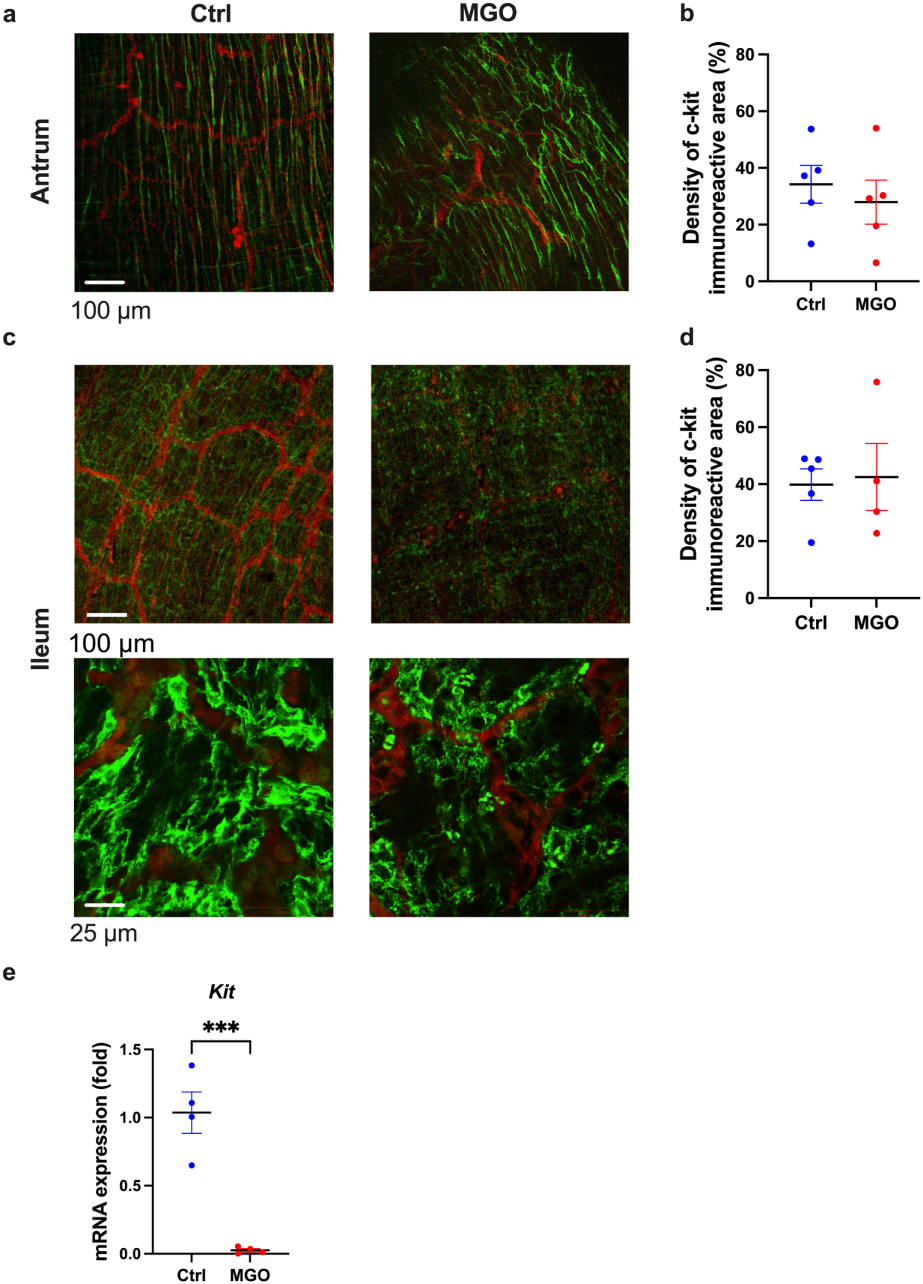
Changes in the ICC morphology in the ileal muscle layer from MGO‐treated mice. (a, c) C‐kit‐positive areas of ICCs of the antrum and ileum stained by immunostaining. The red and green signals indicate the PGP9.5‐positive enteric plexus and c‐kit‐positive ICC (white scale bar = 100 or 25 μm). (b, d) Density of the c‐kit‐positive area. (e) RT‐PCR of the *Kit* mRNA expression to the ileal muscularis. ***p < 0.001 (*n* = 4). Each column shows mean ± SEM.

We considered the second possible cause, i.e., the impairment of the myenteric nerve plexus, PDGFRα‐positive cells, and ICCs downregulates intestinal transit capacity, since these three are largely involved in the contraction of intestinal smooth muscle [21]. First, we measured the PGP9.5‐positive area in the ileal smooth muscle of MGO‐treated mice and found no change compared to the control group (Figure S3). Second, we performed immunofluorescent staining of PDFGRα in the ileal muscle layer of the control and MGO‐treated mice and found no difference in the PDFGRα‐positive areas between them (Figure 5a,b). Furthermore, we obtained similar results for mRNA expression of *Pdgfra* in the muscle layer (Figure 5c), suggesting that the nerve plexus and PDGFRα‐positive cells were not significantly affected. Finally, we evaluated the morphology of ICCs using fluorescent immunostaining for c‐kit in the antrum and ileum of MGO‐treated mice. Although the c‐kit positive area did not change, the ICC shape changed to a granular morphology only in the ileum, indicating ICC atrophy (Figure 6a–d). In addition, the *Kit* mRNA expression was considerably decreased in the intestinal muscle layer of the MGO‐treated mice compared to that in the control mice (Figure 6e). These results suggest the occurrence of some aberrations in the ICCs of the ileum of the MGO‐treated mice.

### 3.5 ICCs Function Was Impaired in the MGO‐Treated Mice

**FIGURE 7:**
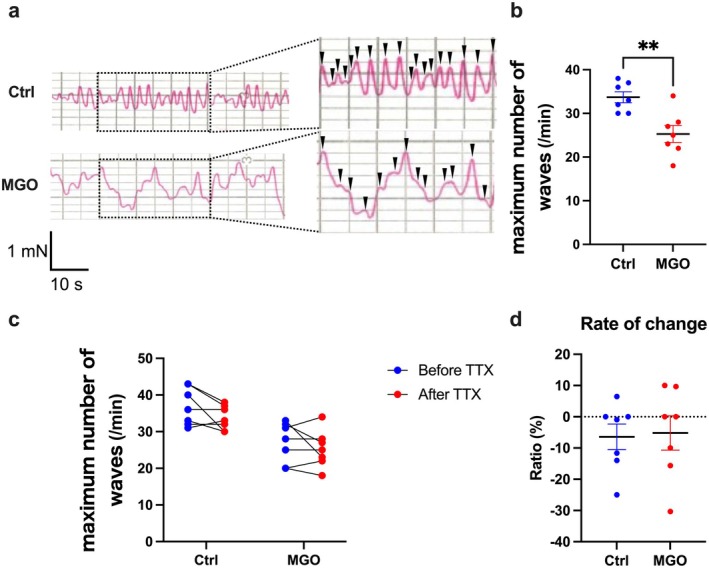
Functional changes in the ICCs in the ileal muscle layer from MGO‐treated mice. (a) Waveform of intestinal motility. (b) Peak number of waves in 20 min. (c) Peak number of waves before and after administration of TTX. (d) Rate of change in the number of wave peaks before and after administration of TTX. **p < 0.01 (*n* = 7). Each column shows mean ± SEM.

The ICCs function was tested in the MGO‐treated mice due to the obvious aberrations in the *Kit* mRNA expression pattern. Spontaneous contractions of the intestinal smooth muscles are governed by ICCs and enteric plexus nerves; thus, the frequency of spontaneous contractions was measured ex vivo (Figure S4). To evaluate the function of ICCs, TTX was added to deactivate the enteric plexus nerve function. Spontaneous rhythmic oscillations occurred in the intestines of the control mice. However, in the intestines of the MGO‐treated mice, the baseline contractile force showed fluctuations and the rhythmic spontaneous contractions changed to an irregular pattern (Figure 7a). The contraction frequency was significantly decreased in the smooth muscles of the MGO‐treated mice compared to that in the control mice after TTX administration (Figure 7a,b). Moreover, we considered that the impact of the nerves on spontaneous contraction would be reflected in the changes before and after TTX treatment. Comparison of the number of contractions before and after TTX treatment revealed no significant difference in the rate of decrease in the number of contractions before and after TTX administration (Figure 7c,d). This means that the myenteric plexus nerves were not affected by MGO, consistent with the data in Figure S3. These data suggest the impaired ICC function in the muscle layers of the MGO‐treated mice.

## 4 Discussion

Gastrointestinal disorders are more likely to occur in patients undergoing PD [7]; however, to date, the underlying mechanism has not been investigated. In this study, we aimed to elucidate the mechanism underlying gastrointestinal disorders in PD patients using mice. The study findings revealed that the gastrointestinal transit capacity was impaired in the MGO‐treated mice, similar to the pattern observed in the patients undergoing PD [7]. Furthermore, inflammation and ICC dysfunction were observed in the intestinal smooth muscles of the MGO‐treated mice. Given that the impaired ICCs function and inflammation in the intestinal tract disturb the gastrointestinal transit function [11, 22], long‐term PD may induce inflammation and ICC dysfunction, which in turn might induce gastrointestinal dysmotility.

Presence of advanced glycation end products (AGEs) in the dialysate, that are glucose metabolites, could be the possible underlying cause of inflammation and ICC dysfunction. Even in the absence of infection or acute peritonitis during PD, AGEs accumulate in the peritoneum and cause injury, thereby promoting inflammation and extracellular matrix deposition [23, 24, 25]. Accordingly, AGEs produced from glucose in the dialysate potentially lead to inflammation and ICC dysfunction in the intestinal muscle layer. The use of inhibitors of AGE receptors in future studies may confirm this possible mechanism.

Similar to the pattern that is observed in the patients undergoing PD, this study revealed that the MGO‐treated mice showed a decreased intestinal transit capacity. This is most likely owing to the inflammation and impairment of ICCs in the smooth muscle of the small intestine but not in the stomach. The muscle layer of the gastric and intestinal walls in mice are approximately 70 and 30 μm thick, respectively [26, 27]. Thus, the harmful effects of AGEs could be absent in the stomach owing to the thickness of the stomach muscle layer. In addition, the small intestine may be more vulnerable to the effects of AGEs as it occupies approximately six times the gastric area in the abdominal cavity [28].

Inflammatory cell infiltration in the duodenum and jejunum was less severe than that in the ileum (Figure 3), suggesting that the gastrointestinal abnormalities in this mouse model occurred mainly in the ileum. This may be attributed to the fact that mice walk on four legs and the ileum is relatively on the abdominal side, while the duodenum is located relatively on the dorsal side. This anatomical difference may cause the dialysate containing MGO to accumulate in the abdomen and contact the ileum more efficiently.

Figure 4 shows an infiltration in the inflammatory cells and an increase in the mRNA expression of the inflammatory mediators 3 h after the administration of the last MGO dose. These results suggest the occurrence of an inflammatory response in the muscle layer; however, no increase in the mRNA expression was observed at 24 and 48 h after the administration of the last MGO dose (Figure 4b,c). Thus, these findings suggest the infiltration of inflammatory cells due to the upregulation of the inflammatory mediators that peaked 3 h after the administration of the last MGO dose and persisted until 48 h, while the severity of inflammation was alleviated around 24–48 h after the administration of the last MGO dose. Although inflammatory cell infiltration was observed at 48 h, mRNA expression was not increased. In general, temporal differences exist between peak mRNA expression and physiological phenomena. We expected that the mRNA expression of various cytokines observed at 3 h would recruit inflammatory cells, and these cells remained for 24–48 h, although their activity diminished. Moreover, the MGO‐treated mice used in this study represent a model of chronic inflammation. As IFN‐γ is not altered in irritable bowel syndrome [29], it is difficult to detect increased mRNA expression of inflammatory cytokines in some chronic inflammatory diseases. Therefore, it is possible that the increase in mRNA expression was less pronounced in our MGO‐treated mice. Nevertheless, the increase in inflammatory cytokine mRNA expression at 3 h, coupled with the infiltration of round inflammatory macrophages at 48 h, supports the conclusion that inflammation was induced.

The enzyme iNOS, which is encoded by *Nos2*, produces nitric oxide (NO) [30], which in turn relaxes the gastrointestinal tract muscles [31]. In the present study, the *Nos2* mRNA expression was not upregulated in the intestinal muscle layer of the MGO‐treated mice (Figure 4b,c). Accordingly, NO might not play a major role in the impaired intestinal transit capacity of the patients undergoing PD. Prostaglandin E_2_ (PGE_2_), a major inflammatory mediator produced from arachidonic acid [32], is an important regulator of gastrointestinal motility via the PGE_2_‐EP2/4 axis [32] and induces relaxation in the colon [33]. Thus, the intestinal contractions might be impaired by PGE_2_‐induced relaxation rather than NO‐induced relaxation [34]. Accordingly, the effects of other inflammatory mediators should be considered.

Immunohistochemical staining revealed c‐kit‐positive areas indicating that the ICCs were not significantly reduced in the intestine of the MGO‐treated mice; however, some c‐kit‐positive images in the ileum displayed a granular morphological appearance without a network form (Figure 6c), which possibly indicates ICC atrophy. The marked decrease in the *Kit* mRNA expression could indicate the reduced proliferative and regenerative capacity of ICCs [35]. In support of the ICC morphological abnormalities, the regular rhythmic spontaneous contractions in the small intestines of the MGO‐treated mice were disrupted, and the frequency of spontaneous contractions was reduced in the presence of TTX. We previously reported morphological abnormalities of ICCs and disrupted spontaneous contractions in various disease models of enteritis [36, 37, 38, 39]. This strongly suggests that ICC dysfunction and the associated morphological changes in the intestines of the MGO‐treated mice could partially be the underlying cause of intestinal dysmotility. In patients undergoing PD, repeated exposure to dialysate may impair the pacemaker function of ICCs as well as their proliferative and regenerative capacities. This might result in the disappearance of healthy ICCs and subsequent impairment of the gastrointestinal transit capacity.

This study has some limitations. The function of ICCs was investigated based on the frequency of spontaneous contractions in the intestinal smooth muscles. Spontaneous intestinal contraction is driven by ICCs, nerves, and smooth muscles [40]. In our experiments, the effect of intestinal smooth muscles on spontaneous contractions could not be excluded despite the use of TTX to block the neuronal effect on contractions. Accordingly, it was not possible to accurately conclude whether the observed decrease in spontaneous contractions (Figure 7) was due to a dysfunction in the ICCs or damage in the smooth muscles. Thus, the accurate measurement of the ICCs function, which requires electrical recording of the pacemaker potentials using the microelectrode method, is recommended to be performed in future studies [39].

NOS nerves play a crucial role in regulating intestinal transit function [19]. Since the PGP9.5‐positive area did not change between controls and MGO‐treated mice in this study, we did not perform a detailed examination of the nerves. However, we cannot exclude the possibility that some injury may be observed when we focus exclusively on the NOS nerve. To definitively rule out decreased contractility resulting from nerve damage, a detailed evaluation is necessary, such as specific staining of NOS nerves by immunostaining and counting nerve junctions. A comprehensive review that focuses on the NOS nerve should be conducted in the future.

In conclusion, this study identified several mechanisms underlying gastrointestinal disorders in patients undergoing PD. These findings could be beneficial for promoting the safe implementation of PD in patients with end‐stage kidney disease.

## Author Contributions

Y.Y. designed the study, main conceptual ideas, and proof outline, and collected the data. T.M. conducted the FITC transit test in the MGO mice, aided in interpreting the results, and supervised the project. Y.Y. wrote the manuscript with support from M.H. and T.M. All authors discussed the results and commented on the manuscript.

## Conflicts of Interest

The authors declare no conflicts of interest.

## Supporting information


**Figure S1.** Colonic transit.
**Figure S2.** mRNA expression of inflammatory mediators in the ileal muscle layer 3 h after vehicle administration.
**Figure S3.** Density of PGP9.5 positive areas in the ileum.
**Figure S4.** Protocol of the frequency of the ex vivo spontaneous contractions.
**Table S1.** Specifications of the animal diet composition.
**Table S2.** The antibodies used in the immunofluorescence staining.
**Table S3.** The used RT‐PCR primers.

## Data Availability

The data that support the findings of this study are available from the corresponding author upon reasonable request.
