# Supplementary material for: Intestinal Dysmotility and Associated Disorders in Intestinal Muscle of Methylglyoxal‐Treated Mice
Source: Neurogastroenterol Motil. 2025 May 2;37(10):e70068. doi: 10.1111/nmo.70068 (PMC12435800; doi:10.1111/nmo.70068)
Supplement: Supplementary file 1 — Figure S1. Colonic transit. Figure S2. mRNA expression of inflammatory mediators in the ileal muscle layer 3 h after vehicle administration. Figure S3. Density of PGP9.5 positive areas in the ileum. Figure S4. Protocol of the frequency of the ex vivo spontaneous contractions. Table S1. Specifications of the animal diet composition. Table S2. The antibodies used in the immunofluorescence staining. Table S3. The used RT‐PCR primers. [file NMO-37-e70068-s001.docx]

Supplementary material

**Supplemental Figure S1**. Colon transit


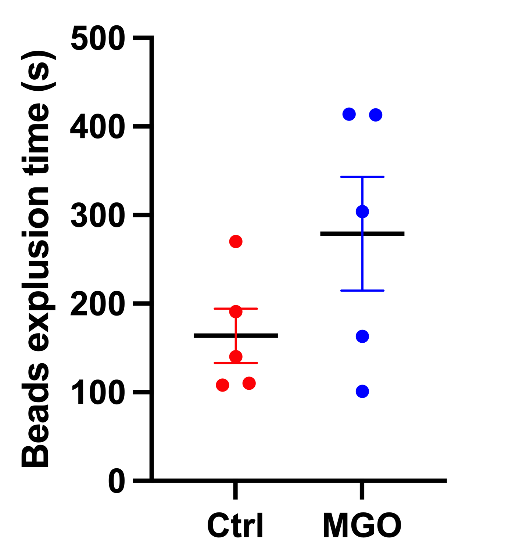


**Supplemental Figure S2**. mRNA expression of *Il1b, Il6, Tnf,* and *Nos2* after the administration of the last dose.


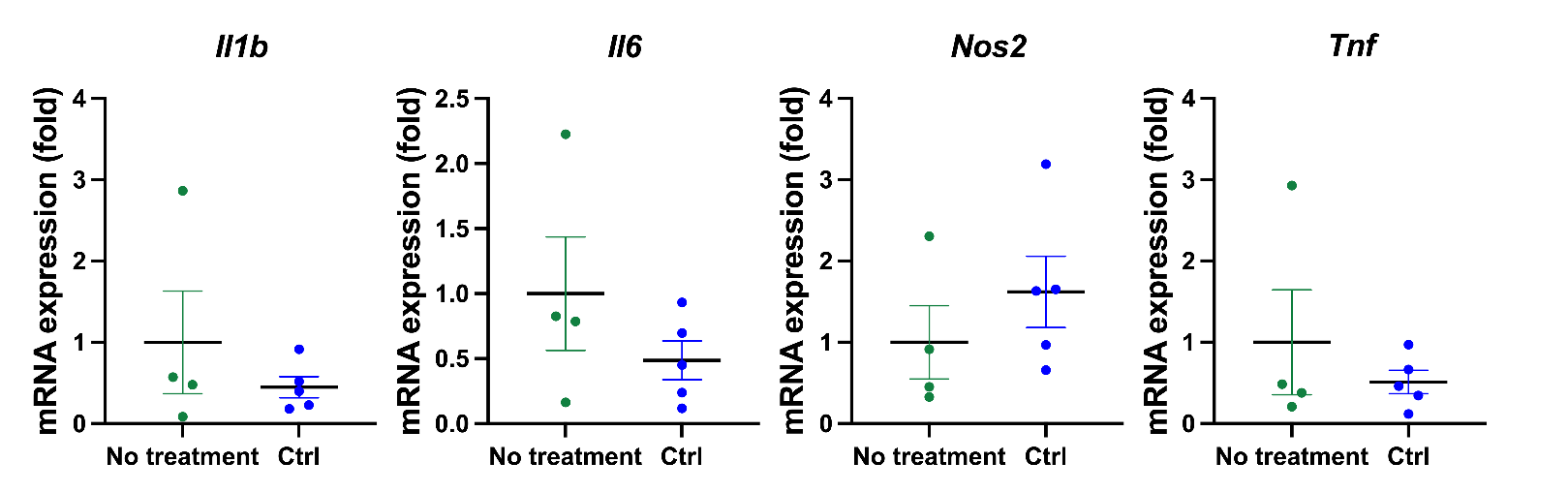


**Supplemental Figure S3**. Density of PGP9.5 positive area in ileum.


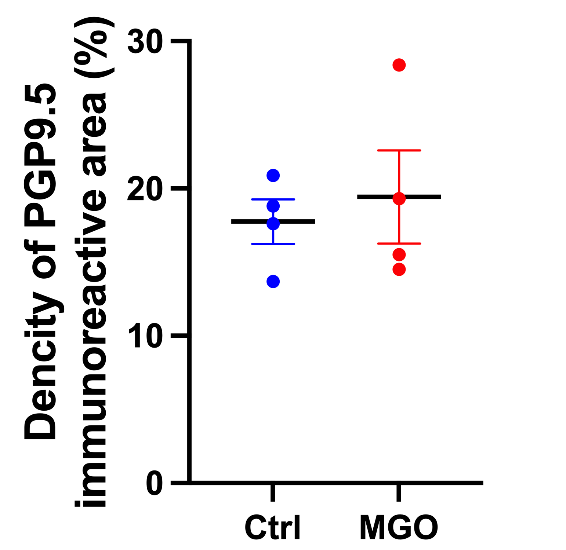


**Supplemental Figure S4**. Protocol of the frequency of the *ex vivo* spontaneous contractions*.*


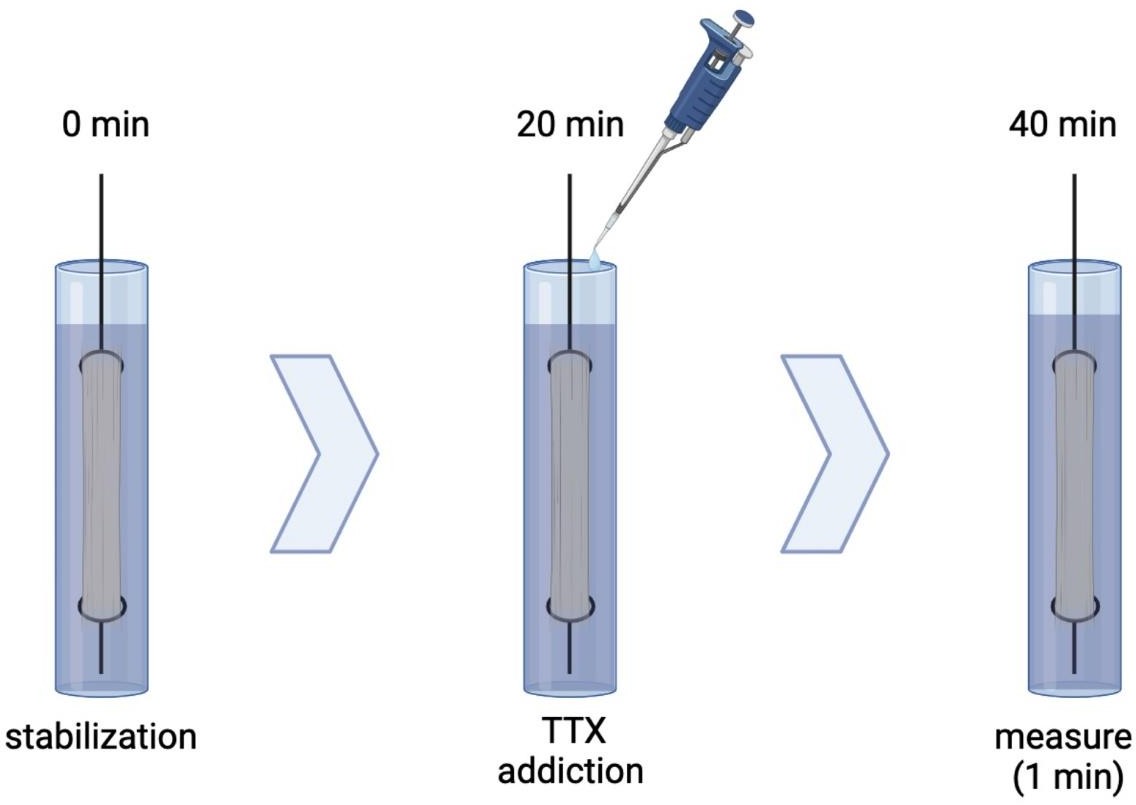


**Supplemental Table S1.** Specifications of the animal diet composition.

| Weight | (g, in 100 g) |
| --- | --- |
| Water | 8.1 |
| Crude protein | 23.2 |
| Crude fat | 4.9 |
| Ash | 5.9 |
| Crude fiber | 3.3 |
| NFE | 54.7 |

**Supplemental Table S2.** The antibodies used in the immunofluorescence staining.

| Primary antibodies | Dilution rate |
| --- | --- |
| Rat anti-mouse CD68 monoclonal antibody (Serotec, SAB1401062-50UG) | 1: 200 |
| Rat anti-mouse CD117 antibody (MyBioSource, MBS673627) | 1: 200 |
| Goat anti-mouse PDGF R alpha antibody (R&D Systems, AF1062) | 1: 400 |
| Rabbit anti-human PGP9.5 polyclonal antibody (Enzo, ADI-905-520-1) | 1: 200 |
| Secondary antibodies | Dilution rate |
| Alexa Fluor® 488 Donkey Anti-rat IgG (Invitrogen, A-21208) | 1: 200 |
| Alexa Fluor® 594 Donkey Anti-rabbit IgG (Invitrogen, R37119) | 1: 200 |
| Alexa Fluor® 594 Donkey Anti-goat IgG (Invitrogen, A-11058) | 1: 400 |

**Supplemental Table S3.** The used RT-PCR primers.

| Name | Forward primers | Reverse primers | Size (bps) |
| --- | --- | --- | --- |
| S18r | AAACGGCTACCACATCCAAG | CCTCCAATGGATCCTCGTTA | 155 |
| Il1b | ATTTTGCATGACACTCTTCACCAC | TAGGCTTGTCTCTGGGTCCTCT | 118 |
| Il6 | CGTGGAAATGAGAAAAGAGTTGTGC | GGTACTCCAGAAGACCAGAGGA | 176 |
| Nos2 | GACGGACCCCAAAAGATGAA | ACAGCTTCTCCACAGCCACA | 145 |
| Pdgfra | TCAGCTGTCTCCTCACAGGG | ACTCTCCCCAACGCATCTCA | 128 |
| Tnf | CAAACCACCAAGTGGAGGAG | GTAGACAAGGTACAACCCATCG | 125 |
